# Optimum dose of spinal ropivacaine with or without single intravenous bolus of S-ketamine during elective cesarean delivery: a randomized, double-blind, sequential dose-finding study

**DOI:** 10.1186/s12884-021-04229-y

**Published:** 2021-11-04

**Authors:** Xiaoyu Zhang, Jianwei Wang, Xiao-Hu An, Yu-Chieh Chao, Yong Bian, Zifeng Xu, Tao Xu

**Affiliations:** 1 Department of Anaesthesiology, the International Peace Maternity and Child Health Hospital, School of Medicine, Shanghai Jiao Tong University, Henshan Road 910, Shanghai, China; 2 Department of Anaesthesiology, Shanghai Children’s Medical Center Affiliated to School of Medicine, Shanghai Jiao Tong University, Shanghai, China

**Keywords:** ED90, Ropivacaine, Post-spinal hypotension, S-ketamine, Caesarean delivery, Post-operative analgesia

## Abstract

**Background:**

Maternal hypotension after spinal anaesthesia occurs at a high rate during caesarean delivery and can lead to adverse maternal or foetal outcomes. The aim of this study was to determine the optimal dose of spinal ropivacaine for caesarean section with or without intravenous single bolus of S-ketamine and to observe the rates of hypotension associated with both methods.

**Methods:**

Eighty women undergoing elective caesarean delivery were randomly allocated into either a ropivacaine only or ropivacaine with intravenous S-ketamine group. If the upper sensory level of the patient reached T6 and the visual analogue scale (VAS) scores remained below 3 points before delivery, the next patient had a 1/9th chance of receiving a lower dose or an 8/9th chance of receiving the same dose as the previous patient. If the patient had VAS scores of more than 2 points or needed an extra epidural rescue bolus before delivery, a higher dose was used for the next patient. The primary outcome was the successful use of spinal ropivacaine to maintain patient VAS score of < 3 points before delivery and the incidence of post-spinal hypotension in both groups. Secondary outcomes included the rates of hypotension-related symptoms and interventions, upper sensory level of anaesthesia, level of sedation, neonatal outcomes, Edinburgh Postnatal Depression Scale scores at admission and discharge, and post-operative analgesic effect. The 90% effective dose (ED90) and 95% confidence interval (95% CI) were estimated by isotonic regression.

**Results:**

The estimated ED90 of ropivacaine was 11.8 mg (95% CI: 11.7–12.7) with and 14.7 mg (95% CI: 14.6–16.0) without intravenous S-ketamine, using biased coin up-down sequential dose-finding method. The rates of hypotension and associated symptoms were significantly lower in S-ketamine group than in the ropivacaine only group.

**Conclusions:**

A spinal dose of ropivacaine 12 mg with a single intravenous 0.15 mg/kg bolus dose of S-ketamine may significantly reduce the risk of hypotension and induce sedation before delivery. This method may be used with appropriate caution for women undergoing elective caesarean delivery and at a high risk of hypotension or experiencing extreme nervousness.

**Trial registration:**

http://www.chictr.org.cn (ChiCTR2000040375; 28/11/2020).

**Supplementary Information:**

The online version contains supplementary material available at 10.1186/s12884-021-04229-y.

## Background

Maternal hypotension is the most common side effect of spinal anaesthesia during caesarean delivery, accounting for 70–83% of all cases [1–3]. It may increase the risk of adverse maternal and foetal outcomes, such as maternal nausea, vomiting, dyspnoea, low Apgar scores, and foetal acidosis [4–7]. Thus, hypotension prevention is paramount to good outcomes. Methods such as intrathecal adjuvant opioid use to reduce the total dose of anaesthetics delivered [8], prophylactic use of vasopressors [4, 9], and the use of intravenous (IV) pre-load colloid liquid [10] have been proposed to reduce the incidence or extent of maternal hypotension after spinal anaesthesia during caesarean delivery.

S-ketamine is an S+ isomer of ketamine that is twice as potent as ketamine [11]; it achieves sedation, analgesia, and sympathetic activation [12] with a relatively low risk of side effects to mothers or infants [13], making it an optimum choice as an IV adjuvant for spinal anaesthesia during caesarean delivery. To reduce the risk of psychomimetic reactions, S-ketamine administration was limited to an ultra-low single dose in the present study.

We hypothesized that a single ultra-low IV bolus of S-ketamine may reduce the total need for spinal ropivacaine and thus reduce the incidence of post-spinal anaesthesia hypotension during caesarean delivery. Finally, 90% effective doses (ED90) of spinal ropivacaine with and without a single IV bolus of S-ketamine were estimated, using the biased coin up-down (BCUD) sequential dose-finding method; the incidence of post-spinal anaesthesia hypotension was observed.

## Methods

This was a prospective, double-blind, randomised, BCUD-based, sequential dose-finding study approved by China Ethics Committee of Registering Clinical Trials, West Hospital, Sichuan University, Sichuan, China on 29 September 2020 (Chairperson Taixiang Wu, Ethical No.: ChiECRCT20200301) and registered at http://www.chictr.org.cn (ChiCTR2000040375; 28/11/2020). A total of 85 women, aged 22–40 years, and with a full-term (> 37 weeks of gestation) singleton pregnancy, the American Society of Anaesthesiologists (ASA) physical status II, and undergoing an elective caesarean delivery were recruited during January 2021 and April 2021 at the International Peace Maternity and Child Health Hospital. After providing signed informed consent, the women were allocated by a research assistant to one of two groups, using a random number generator in SPSS for Windows version 18.0 (SPSS Inc., Chicago, IL, USA), specifically, ropivacaine alone (R) and ropivacaine with intravenous S-ketamine (RS) groups. All patients were blinded to group allocation.

Exclusion criteria were as follows: contraindications to combined spinal and epidural (CSE) anaesthesia; the ASA score of III–IV; allergy to ketamine, S-ketamine, or ropivacaine; history of gastroesophageal reflux, full stomach, placenta previa, placental abruption, pre-existing or pregnancy-induced hypertension, preeclampsia, cardiovascular or cerebrovascular disease; foetal distress or abnormalities; multiple gestations; refusal to participate, and emergent caesarean delivery. Due to differences in delivery time and uterine contraction pain levels after surgery, women who had previously undergone a caesarean section and multiparas were excluded.

Patients were recruited for the study on the day before surgery in the obstetrical wards, when they were asked to complete the Edinburgh Postnatal Depression Scale (EPDS), which is a screening instrument that helps detect women at risk for depression in the perinatal period [14]. All patients were instructed to fast from midnight on the day of surgery. After entering the operating room, an IV line was established with an 18G IV catheter in the left forearm, and lactated Ringer’s solution was infused at a rate of 1 ml.min^− 1^ to maintain the vein open.

Routine monitoring was performed continuously, including electrocardiography, non-invasive blood pressure, and pulse oximetry. Systolic blood pressure (SBP), heart rate (HR), and pulse oximetry were assessed every 2 min. The first two resting SBP and HR measurements obtained in the supine position were recorded, and their average values were treated as the baseline values. If the baseline SBP was of > 140 mmHg, the patient was excluded from the study due to hypertension.

The CSE puncture was performed routinely at the level of L3–4 by an anaesthesiologist with more than 15 years of experience, while the patient was in the right lateral decubitus position. A 17G Tuohy needle was used to perform the epidural puncture with a paramedian approach. After identifying an entrance into the epidural space, a 27G Whitacre needle was inserted through the epidural needle. Once the cerebrospinal fluid was detected, an isobaric ropivacaine dose of 0.75% was injected through the Whitacre needle. After injecting the ropivacaine, an epidural catheter was inserted into the epidural space by advancing it 3 cm through the Tuohy needle. The patient was immediately moved to the supine position with the left uterine displacement created by placing a wedge under the right hip.

In addition, an infusion of 1 ml.kg^− 1^.h^− 1^ lactated Ringer’s solution was administered until delivery. A rescue 5-ml bolus of 2% lidocaine was administered through the epidural catheter 2 min before the surgery for patients that could still feel the cold sensation of ice being placed on the skin below the level of T6 and those that complained of pain and whose VAS score was of > 2 points during the surgery. A 5-ml IV dose of 0.15 mg.kg^− 1^ S-ketamine with normal saline or 5 ml normal saline solution was administered with a 5-ml syringe 1 min before the surgery, depending on group assignment. Hypotension was defined as SBP of < 80% of the baseline value. A rescue dose of 50 μg phenylephrine was administered whenever hypotension was detected. Bradycardia was defined as HR of < 50 beats per minute (bpm) and treated with 0.5 mg atropine. The level of sedation was assessed using a 5-point scale (1 = agitated; 2 = alert; 3 = calm; 4 = drowsy; 5 = asleep) [15].

Immediately after delivery, 1 ml of umbilical vein blood was collected by the obstetrician, and blood gas assessments were performed, using a blood gas analyser (iSTAT1 Analyzer MN:300-G, Abbott Point of Care Inc., USA) with an iSTAT CG7+ test cartridge. A patient-controlled intravenous analgesia (PCIA) pump (AM3300, ACE MEDICAL EQUIPMENT INC., Korea) was connected to the patient, and a 2-mL PCIA bolus was given at the end of the surgery; meanwhile, the patient was taught how to use the PCIA pump. The PCIA fluid consisted of 10 mg hydromorphone and 90 ml normal saline at the total volume of 100 ml. A 2-ml background infusion and a 2-ml patient-controlled analgesia (PCA) bolus was set to a 15-min lockout time. The patient was followed-up at bedside for the post-operative analgesic effect and recovery of lower limb mobility 24 and 48 h after surgery. The EPDS was administered at discharge.

In both RS and R groups, a research spinal dose of ropivacaine in a 5-ml syringe was prepared and given to the patient through the Whitacre needle by a specialist anaesthesiologist with over 15 years of experience, who knew the exact administered dose. The remaining anaesthesiologists and all patients were blinded to the administered doses.

The spinal ropivacaine dose of 12 mg was used for the first patient in both groups, based on findings from Tang et al. [16] and Mei et al. [17]. The dose administered to each subsequent patient was determined by the response of the immediately preceding patient. Satisfactory anaesthetic effect was defined as the upper sensory level of spinal anaesthesia not below T6 before surgery and the VAS score of ≤2 points before delivery. Provided a satisfactory anaesthetic effect was achieved, the spinal dose of ropivacaine was considered suitable, and the next patient was 1/9-times as likely to receive a lower dose (decreased by 1.5 mg) or 8/9-times as likely to receive the same dose as the previous patient. When satisfactory anaesthetic effect was not achieved, the following patient received a dose that was increased by 1.5 mg. Minimum and maximum doses of spinal ropivacaine were 9 mg and 18.5 mg, respectively. The dose assignment was prepared by a statistician, using the BCUD function in Microsoft Excel 2016; only the specialist anaesthesiologist had access to this information, ensuring the double-blind nature of this study.

The primary outcomes were satisfactory anaesthetic effect until delivery and the incidence of hypotension. Secondary outcomes included maternal hypotension-related symptoms and their management, upper sensory level, total co-load IV fluid volume, blood loss volume, level of sedation during surgery, recovery of lower limb mobility, analgesic effect 24 and 48 h after surgery, and EPDS scores at discharge; neonatal outcomes included induction-delivery interval, umbilical vein blood gas values (including pH, pO_2_, pCO_2_, base excess [BE], SO_2_, and lactate levels), neonatal weight, Apgar scores measured at 1 min and 5 min post-delivery, and the incidence of neonatal intensive care unit admission. Maternal demographic characteristics such as age, weight, height, gestational weeks, gravida, para, baseline SBP, baseline HR, and EPDS scores at admission were recorded. All primary and secondary outcomes were observed by anaesthesiologists blinded to patient group and dose assignments.

### Statistical analysis and sample size calculation

This dose finding study was based on BCUD design and simulation studies that suggest that a sample size of 20–40 patients may provide stable estimates of the target dose for most realistic cases [18]. The present study included 40 patients per group.

The ED90 was defined as the spinal dose of ropivacaine associated with a 90% anaesthetic success rate and was estimated, using the isotonic regression method [18]. The corresponding 95% confidence interval (CI) was obtained, using the bias-corrected percentile method with 2000 bootstrap replications [19]. Isotonic regression and bootstrapping analyses were performed in R software version 3.4.4. (R Foundation for Statistical Computing, Vienna, Austria).

Demographic characteristics and secondary outcome estimates were reported as means ± standard deviations, medians (interquartile range), and counts and proportions. Parametric data were analysed with the t-test; nonparametric data were analysed with the Mann-Whitney test. Proportions were compared, using the Chi-square and Fisher exact tests, as suitable. The peri-operative haemodynamic parameters were assessed by multiple t-tests and estimated with a two-stage linear step-up procedure of Benjamini, Krieger, and Yekutieli, with Q = 1%. Statistical comparisons were made, using SPSS for Windows version 18.0 (SPSS Inc., Chicago, IL, USA) and GraphPad Prism 8 for windows (GraphPad Software, San Diego, California, USA). Statistical significance was defined as *p*-values of < 0.05.

## Results

**Fig. 1:**
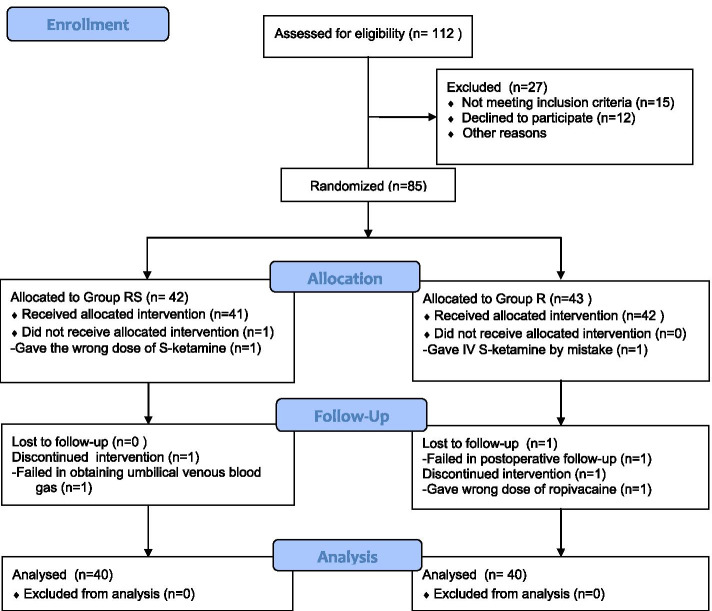
Flow chart of the study

The patient recruitment process is presented in Fig. 1. Data from 40 patients per group were included in the analysis. Maternal characteristics were similar between the groups and are presented in Table 1.

**Table 1:** Maternal characteristics

Characteristics	Group RS (*n* = 40)	Group R (n = 40)	*P* value
Age, years	32.8 ± 5.0	31.6 ± 3.3	0.196
Weight, kg	69.7 ± 7.5	71.4 ± 8.9	0.359
Height, cm	162.8 ± 4.9	161.0 ± 4.2	0.080
Gestational weeks	39.1 ± 0.9	38.9 ± 1.1	0.353
Gravida, n	1(1–2)^a^	1(1–1)^a^	0.096
Para, n	0(0–0)^a^	0(0–0)^a^	1.000
Baseline HR, bpm	84.3 ± 13.1	89.9 ± 14.4	0.069
Baseline SBP, mmHg	120.1 ± 11.6	124.0 ± 9.8	0.104
EPDS scores at admission	2.9 ± 2.3	3.5 ± 2.5	0.291

Values are mean ± SD or ^a^median (IQR)

*HR* heart rate, *SBP* systolic blood pressure, *EPDS* Edinburgh Postnatal Depression Scale, *IQR* interquartile range

**Fig. 2:**
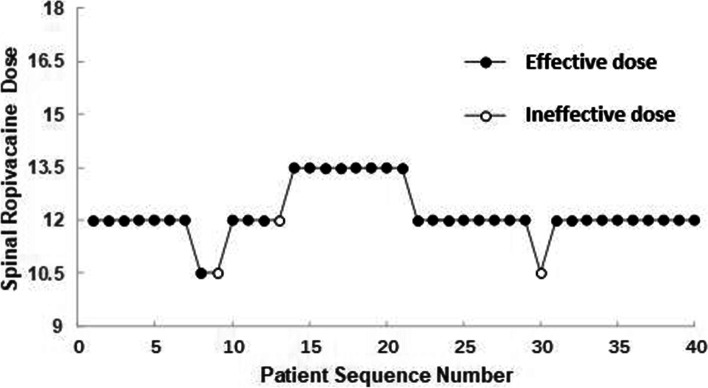
The patient allocation sequence and the response to the assigned dose of spinal ropivacaine in RS group. The patient sequence number (X-axis) is the order of patient exposures using the BCUD design. The assigned dose levels are presented on Y-axis. An effective dose is denoted by a solid circle, while an ineffective one is denoted by a hollow circle. BCUD, biased coin up and down

**Fig. 3:**
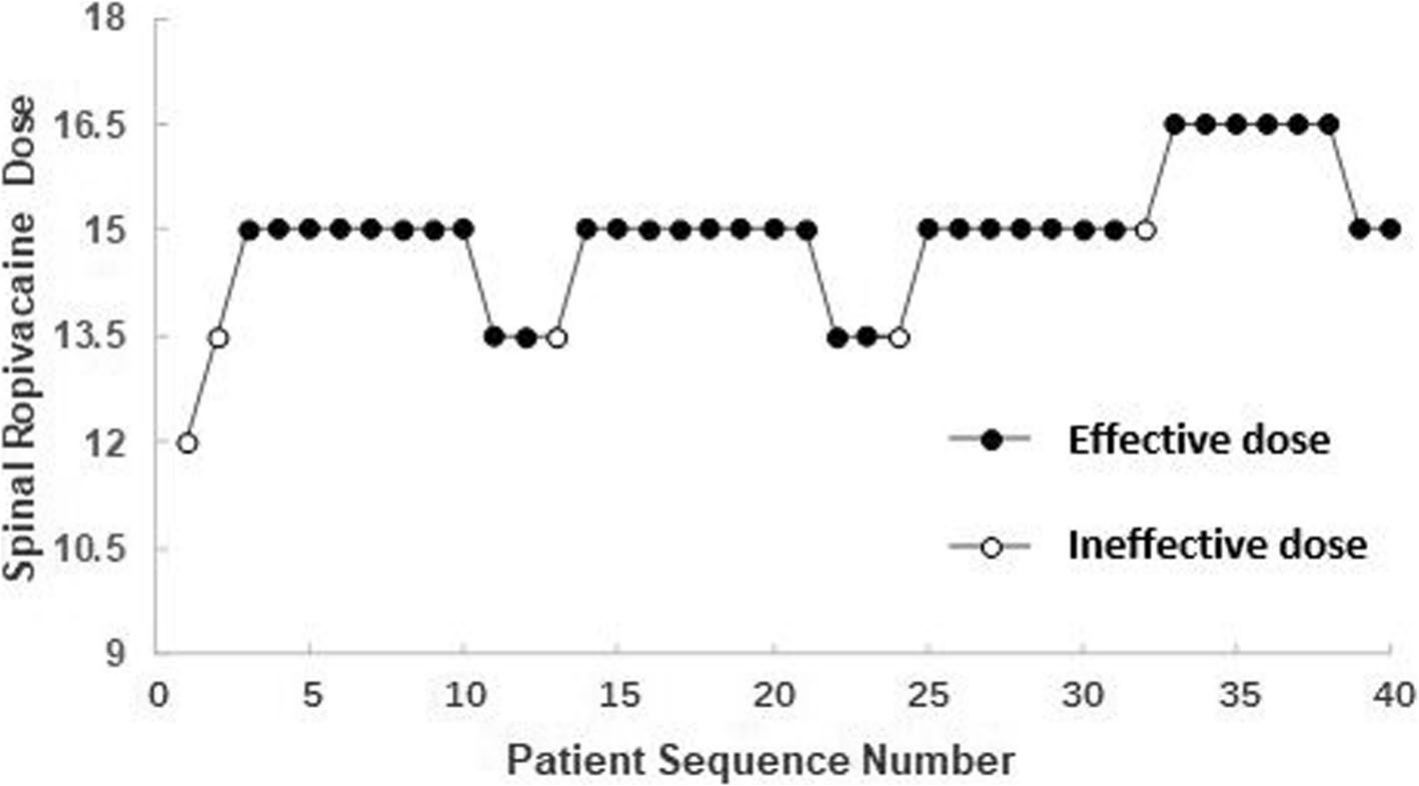
The patient allocation sequence and the response to the assigned dose of spinal ropivacaine in R group. The patient sequence number (X-axis) is the order of patient exposures using the BCUD design. The assigned dose levels are presented on Y-axis. An effective dose is denoted by a solid circle, while an ineffective one is denoted by a hollow circle. BCUD, biased coin up and down

Figures 2 and 3 present the sequence of effective and ineffective responses to spinal ropivacaine dose in consecutive patients in the R and RS groups, respectively. The observed and pooled-adjacent-violators algorithm-adjusted response rates associated with each spinal ropivacaine dose in the R and RS groups are presented in Tables 2 and 3, respectively. The ED90 value of spinal ropivacaine with IV S-ketamine was 11.8 mg (95% CI: 11.7 to 12.7) and the corresponding value without IV S-ketamine was 14.7 mg (95% CI: 14.6 to 16.0). The estimated median drug saving percentage for spinal ropivacaine between the groups was 19.7% (95% CI: 19.0 to 26.2%).

**Table 2:** Observed and PAVA-Adjusted Response in Group RS

Assigned Dose (mg)	Number of Successes	Number of Patients	Observed Response Rate (%)	PAVA-adjusted Response Rate(%)
10.5	1	3	0.333	0.333
12	28	29	0.966	0.966
13.5	8	8	1.000	1.000

PAVA-adjusted response rates were estimated using the weighted isotonic regression method

*PAVA* pooled-adjacent-violators algorithm

**Table 3:** Observed and PAVA-Adjusted Response in Group R

Assigned Dose (mg)	Number of Successes	Number of Patients	Observed Response Rate (%)	PAVA-adjusted Response Rate(%)
12	0	1	0.000	0.000
13.5	4	7	0.571	0.571
15	25	26	0.962	0.962
16.5	6	6	1.000	1.000

PAVA-adjusted response rates were estimated using the weighted isotonic regression method

*PAVA* pooled-adjacent-violators algorithm

Maternal outcomes are presented in Table 4. The incidence of hypotension was lower in the RS group than in the R group (5% vs. 65%, *P* < 0.001). The dose of rescue phenylephrine bolus administered (0 mg [0 to 0] vs. 50 mg [0 to 50], *p* < 0.001), and the rates of bradycardia (0% vs. 15%, *P* = 0.026), dyspnoea (0% vs. 42.5%, *P* < 0.001), nausea (0% vs. 37.5%, P < 0.001), and vomiting (0% vs. 35%, *P* < 0.001) were lower in the RS group than in the R group. The incidence of symptoms associated with S-ketamine such as visceral traction pain (7.5% vs. 45%, *P* < 0.001) was lower in the RS group than in the R group; in contrast, the rates of dreaming (62.5% vs. 0%, *P* < 0.001) and dizziness (52.5% vs. 2.5%, P < 0.001) were higher in the RS group than in the R group. The upper sensory level of spinal anaesthesia was lower in the RS group than in the R group (T5 [4 to 5.75] vs. T4 [3 to 4], *P* < 0.001). The levels of sedation during delivery and 5 min after delivery were significantly different between two groups (*P* < 0.001, *P* = 0.001, respectively).

**Table 4:** Maternal outcomes

Outcomes	Group RS (n = 40)	Group R (n = 40)	*P* value
Peri-operative outcomes			
Induction-IV S-ketamine or NS interval, min	6.8 ± 1.5	7.0 ± 1.8	0.685
Hypotension, %	2(5)	26(65)	< 0.001
Rescue phenylephrine bolus dose	0(0–0)^a^	50(0–50)^a^	< 0.001
Hyoxemia,%	1(2.5)	4(10)	0.359
Bradycardia, %	0(0)	6(15)	0.026
Dyspnea, %	0(0)	17(42.5)	< 0.001
Nausea, %	0(0)	15(37.5)	< 0.001
Vomiting, %	0(0)	14(35)	< 0.001
Shiver, %	0(0)	0(0)	1.000
Visceral traction pain,%	3(7.5)	18(45)	< 0.001
Dreaming,%	25(62.5)	0(0)	< 0.001
Blurred vision,%	0(0)	0(0)	1.000
Dizziness, %	21(52.5)	1(2.5)	< 0.001
Upper sensory level	T5(4–5.75)^a^	T4(3–4)^a^	< 0.001
Total co-load IV fluid volume, ml	830 ± 194	849 ± 221	0.679
Blood loss volume, mL	192.8 ± 25.6	207.0 ± 28.5	0.021
Level of sedation before delivery (score:1/2/3/4/5)	0/0/1/0/39	12/5/23/0/0	< 0.001
Level of sedation 5 min after delivery (score:1/2/3/4/5)	0/0/32/8/0	3/0/37/0/0	0.001
Level of sedation at the end of surgery (score:1/2/3/4/5)	0/0/40/0/0	0/2/38/0/0	0.494
Surgery time, min	40.6 ± 9.2	42.2 ± 8.4	0.422
Post-operative outcomes			
Duration of first PCA, min	484.3 ± 325.6	212.2 ± 61.4	< 0.001
Recovery of lower limb mobility, min	119.3 ± 31.7	152.1 ± 40.0	< 0.001
Resting VAS at 24H	1.4 ± 1.1	3.4 ± 0.9	< 0.001
Mobile VAS at 24H	3.8 ± 1.3	5.9 ± 1.3	< 0.001
PCA times during 24H	1.7 ± 1.8	2.2 ± 2.3	0.005
Resting VAS at 48H	0.7 ± 0.8	1.8 ± 0.7	< 0.001
Mobile VAS at 48H	2.2 ± 1.1	3.4 ± 1.1	< 0.001
PCA times during 24-48H	0.4 ± 0.9	0.7 ± 0.8	0.123
EPDS scores at discharge	2.3 ± 2.5	4.1 ± 3.0	0.004

Values are n(%), mean ± SD, actual numbers or ^a^median (IQR)

*IV* intravenous, *PCA* patient controlled analgesia, *EPDS* Edinburgh Postnatal Depression Scale, *IQR* interquartile range

Moreover, the duration of the first PCA was longer in the RS group than in the R group (212.2 ± 61.4 vs. 484.3 ± 325.6 min, *P* < 0.001). The recovery of lower limb mobility required less time in the RS group than in the R group (119.3 ± 31.7 vs. 152.1 ± 40.0 min, P < 0.001). Resting and mobile VAS scores at 24 h (1.4 ± 1.1 vs. 3.4 ± 0.9 points, P < 0.001 and 3.8 ± 1.3 vs. 5.9 ± 1.3 points, P < 0.001, respectively) and 48 h (0.7 ± 0.8 vs. 1.8 ± 0.7 points, P < 0.001 and 2.2 ± 1.1 vs. 3.4 ± 1.1 points, *P* < 0.001, respectively) were lower in the RS group than in the R group. The PCA time during 24 h was lower in the RS group than in the R group (1.7 ± 1.8 vs. 2.2 ± 2.3 times, *P* = 0.005). The EPDS scores at discharge were lower in the RS group than in the R group (2.3 ± 2.5 vs. 4.1 ± 3.0 points, *P* = 0.004).

Neonatal outcomes are presented in Table 5. The pH value (7.37 ± 0.03 vs. 7.35 ± 0.05, *P* = 0.047) was different between the groups. The levels of PO_2_ (30.9 ± 5.1 mmHg vs. 25.2 ± 6.2 mmHg, *P* < 0.001), BE (− 2.0 ± 1.5 vs − 4.0 ± 1.3 mEq.l^− 1^, P < 0.001), and SO_2_ (57.8 ± 11.2% vs. 43.1 ± 16.2%, P < 0.001) were higher, and those of lactate (1.5 ± 0.3 vs. 1.8 ± 0.4 mmol.l^− 1^, *P* = 0.003) were lower in the RS group than in the R group.

**Table 5:** Neonatal outcomes

Outcomes	Group RS (n = 40)	Group R (n = 40)	*P* value
Induction-delivery interval, min	13.9 ± 3.5	14.9 ± 3.7	0.190
UV analysis			
pH	7.37 ± 0.03	7.35 ± 0.05	0.047
PO_2_, mmHg	30.9 ± 5.1	25.2 ± 6.2	< 0.001
PCO_2_, mmHg	40.0 ± 4.1	39.9 ± 7.1	0.931
BE, mEq/L	−2.0 ± 1.5	−4.0 ± 1.3	< 0.001
SO_2_, %	57.8 ± 11.2	43.1 ± 16.2	< 0.001
Lactate, mmol/L	1.5 ± 0.3	1.8 ± 0.4	0.003
Neonatal Weight, g	3477 ± 404	3448 ± 396	0.747
Apgar Scores at 1 min	10(10–10)^a^	10(10–10)^a^	1.000
Apgar Scores at 5 min	10(10–10)^a^	10(10–10)^a^	1.000
NICU admission	2(5)	5(12.5)	0.432

Values are n(%), mean ± SD or ^a^median (IQR). UV, umbilical vein

**Fig. 4:**
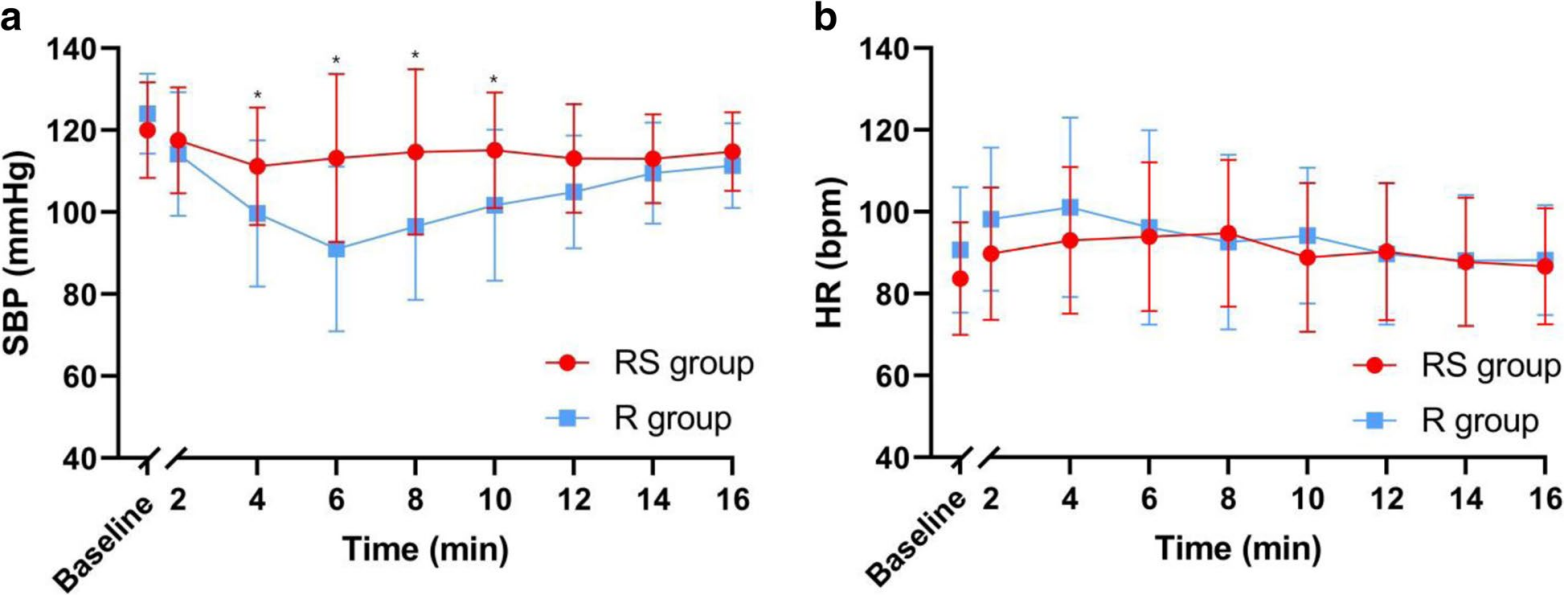
Serial changes of haemodynamic over time after the spinal anaesthesia of two groups. The red circles and their error bars represent the means and SDs of SBP and HR over times of 40 patients in RS group; The blue squares and their error bars represent the means and SDs of SBP and HR over times of 40 patients in R group. **a** Serial changes of SBP over time. **b** Serial changes of HR over time. *Significant difference between the two groups were assessed by multiple t test and determined by Two-stage linear step-up procedure of Benjamini, Krieger and Yekutieli, with Q = 1%. SBP, systolic blood pressure; HR, heart rate; bmp, beats per minute

Serial changes of SBP and HR values over time after spinal anaesthesia administration are presented in Fig. 4. The SB*P* values at 4, 6, 8, and 10 min (*P* = 0.004, < 0.001, < 0.001, < 0.001, respectively) were higher in the RS group than in the R group. Although HR values at 2 and 4 min (*P* = 0.035 and 0.028, respectively) were higher in the R group than in the RS group, two-stage linear step-up procedures revealed no between-group difference in HR values.

## Discussion

In this study, the ED90 of spinal ropivacaine administered with S-ketamine was 11.8 mg (95% CI: 11.7 to 12.7); the corresponding value without S-ketamine was 14.7 mg (95% CI: 14.6 to 16.0), derived with the BCUD method and isotonic regression. The present findings suggest that a single IV S-ketamine bolus of 0.15 mg.kg^− 1^ may help reduce spinal ropivacaine consumption by 19.7% (95% CI: 19.0 to 26.2%), helping reduce the incidence of hypotension and hypotension-related adverse effects after spinal anaesthesia.

Several methods have been proposed to reduce the incidence or severity of maternal hypotension after spinal anaesthesia during caesarean delivery, including the use intrathecal adjuvant opioids to decrease the total dose of  local anaesthetics used [8], prophylactic use of vasopressors [4, 9], and IV pre-load colloid liquid administration [10]. Zieleskiewicz et al. [1] and Yao et al. [3] used B ultrasound, Xu et al. [20] and Toyama et al. used [21] perfusion index of peripheral vessels to predict the risk of hypotension to enable early and individualised interventions. We believe that the fundamental approach to reduce the incidence of post-spinal anaesthesia hypotension is to control the upper anaesthesia level before delivery, while lowering intrathecal drug use and ensuring satisfactory anaesthetic effect. The present findings suggest that an ultra-low single IV bolus dose of S-ketamine may meet these criteria.

S-ketamine, the pure dextrorotatory enantiomer of ketamine, is used in clinical analgesia and anaesthesia. The main effects of S-ketamine are mediated by non-competitive inhibition of the N-methyl-D-aspartate receptor; the compound also interacts with opioid receptors, monoamine receptors, and adenosine receptors [22]. It is associated with dissociation (ability to communicate with the patient is retained to a certain degree), quick onset, absence of breathing function depression, and circulatory stability in shock patients, making it popular among emergency and intensive care medicine practitioners [22]. In addition, it is relatively safe for infants and children [13], making S-ketamine a suitable candidate for an adjuvant IV drug during caesarean delivery with spinal anaesthesia. The present study findings suggest that an ultra-low single IV bolus dose of S-ketamine may induce sleep, decrease visceral traction pain from surgical interventions, and eliminate tension in patients that undergo caesarean delivery without increasing the risk of adverse events.

Furthermore, it has been demonstrated that a low infusion rate of S-ketamine may reduce opioid consumption and the extent of postpartum delirium [23, 24]. In this study, the total dose of S-ketamine was even lower than that previously examined [23, 24], while the post-operative analgesic effect of S-ketamine and its interactions with opioids were maintained; no case of postpartum delirium or agitation was observed in the first 48 h. However, the rates of dreaming and dizziness were higher among the patients that received S-ketamine than among those who did not. Most dreams were associated with high-speed movements; patients experienced the feeling of being shuttled in space. Despite short duration of these side effects and their perceived acceptability among the affected patients, we recommend that patients offered this treatment are carefully briefed ahead of surgery, so they can make an informed decision.

The faster recovery of maternal lower limb mobility in the RS group was an unexpected finding, likely indicating the contribution of the lower spinal dose of ropivacaine. Satisfactory analgesic effect and faster recovery of lower limb mobility may be direct or indirect results of the ultra-low dose of S-ketamine, corresponding to enhanced recovery after surgery.

Anti-depressive effects of ketamine and S-ketamine have been reported in both human and animal studies [25–27]. However, studies on the impact of S-ketamine on postpartum depression are rare. Xu et al. [28] demonstrated that an ultra-low single bolus dose of ketamine does not prevent postpartum depression. Brexanolone infusion is currently the standard treatment for post-partum depression [29]. In this study, an ultra-low single IV bolus of S-ketamine was used after spinal anaesthesia; the EPDS was administered at admission and discharge. The EPDS scores were similar in both groups at admission; however, at discharge, these scores were lower in the patients that did than in those that did not receive S-ketamine, suggesting S-ketamine’s anti-depressive effects in the postpartum population. However, satisfactory post-operative analgesic effect may have contributed to the lower EDPS scores; further studies are required to validate the anti-depressive effect of S-ketamine in this population.

In the present study, the bolus dose of S-ketamine was 0.15 mg.kg^− 1^. Sen et al. [30] and Menktit et al. [31] previously reported that IV ketamine dose of 0.15 mg.kg^− 1^ may produce satisfactory analgesia, while reducing the consumption of post-operative analgesics. Given the 2:1 potency ratio of ketamine and S-ketamine [11], the dose of S-ketamine should be approximately 0.075 mg.kg^− 1^. We tested this lower dose of S-ketamine in preliminary experiments, failing to induce sleep in most patients; consequently, the dose of 0.15 mg.kg^− 1^ was used, with only one patient failing to achieve sleep; this patient was sedated in the present study. Moreover, patients undergoing elective caesarean delivery without gastroesophageal reflux or full stomach were included in this study; thus, neither reflux nor aspiration were observed in any of the study patients. Although the coughing, gag, and swallowing reflexes remained functional after the administration of S-ketamine [22], the use of S-ketamine in patients with full stomach or gastroesophageal reflux disease is unsuitable. Finally, the first contact between mother and neonate is important, promoting parent-child bonding and mother’s willingness to breast-feed. Although some patients remained drowsy 5 min after delivery, all patients with IV S-ketamine in the present study could be awakened at that time; all patients communicated with the neonates and were clear-headed at the end of surgery.

This study has some limitations. First, in this study, both patients and anaesthesiologists were blinded to the spinal dose of ropivacaine administered to each group. However, both the patients and attending anaesthesiologist could identify group assignment based on the hypnotic nature of S-ketamine; however, the resulting biases are unlikely to have influenced the presented findings, as all outcomes were based on separate patient and observer reports. Second, the anti-depressive effect of S-ketamine postpartum was evaluated by the EPDS after post-operative days 4–5, by which point most patients had not recovered either physically or mentally from the impact of caesarean section. Future studies should assess the degree of postpartum depression after ≥6 weeks in this population to comprehensively evaluate the anti-depressive effects of S-ketamine postpartum. Third, the low incidence of maternal hypotension and its related side effects was likely due to the decreased dose of spinal ropivacaine, associated with the use of S-ketamine. Although the effect of stimulating the sympathetic nervous system by S-ketamine has been shown in several previous studies, herein, we could not distinguish between the effect of spinal ropivacaine dose reduction and that of S-ketamine use. Establishing whether an ultra-low bolus dose of S-ketamine increases maternal BP after spinal anaesthesia requires further research. Maternal hypotension tends to occur within 5–10 min after spinal anaesthesia, and if S-ketamine is proved to be able to increase maternal BP, it has the potential to be used earlier in the process than the time point chosen in this study. However, additional evidence is required before this recommendation can be made. Finally, the present study sample was small; large studies are required to validate the safety and side effects of IV S-ketamine for women undergoing elective caesarean delivery.

## Conclusion

The ED90 of spinal ropivacaine was 11.8 mg (95% CI: 11.7 to 12.7) with and 14.7 mg (95% CI 14.6 to 16.0) without S-ketamine. An ultra-low single IV bolus dose of S-ketamine may reduce the required dose of spinal ropivacaine by 19.7% (95% CI: 19.0 to 26.2%), helping reduce the incidence of hypotension and associated symptoms, and helping achieve sedation during caesarean delivery. A spinal ropivacaine of 12 mg with IV S-ketamine bolus of 0.15 mg.kg^− 1^ may be used with caution for patients at high risk of hypotension or experiencing extreme nervousness.

## Supplementary Information

**Additional file 1.**

## Data Availability

All data generated or analyzed during this study are included in this published article (and its supplementary information files).

## Abbreviations

VAS: Visual analogue scale
ED90: 90% effective dose
95% CI: 95% confidence interval
IV: Intravenous
BCUD: Biased coin up-down
ASA: American society of anaesthesiologists
CSE: Combined spinal and epidural
EPDS: Edinburgh Postnatal Depression Scale
SBP: Systolic blood pressure
HR: Heart rate
bpm: Beats per minute
UV: Umbilical vein
PCIA: Patient controlled intravenous analgesia
PCA: Patient controlled analgesia
PAVA: Pooled-adjacent-violators algorithm
NICU: Neonatal intensive care unit
IQR: Interquartile range

## References

[1] Zieleskiewicz L, Noel A, Duclos G (2018). Can point-of-care ultrasound predict spinal hypotension during cesarean section? A prospective observational study. Anesthesia.

[2] Orbach-Zinger S, Ginosar Y, Elliston J (2012). Influence of preoperative anxiety on hypotension after spinal anesthesia in women undergoing cesarean delivery. Br J Anaesth.

[3] Yao SF, Zhao YH, Zheng J (2021). The transverse diameter of right common femoral vein by ultrasound in the supine position for predicting post-spinal hypotension during cesarean delivery. BMC Anesthesiol.

[4] Xu T, Zheng J, An XH (2019). Norepinephrine intravenous prophylactic bolus versus rescue bolus to prevent and treat maternal hypotension after combined spinal and epidural anesthesia during cesarean delivery: a sequential dose-finding study. Ann Transl Med.

[5] Kinsella SM, Carvalho B, Dyer RA (2018). International consensus statement on the management of hypotension with vasopressors during cesarean section under spinal anesthesia. Anesthesia.

[6] Onwochei DN, Ngan Kee WD, Fung L (2017). Norepinephrine intermittent intravenous boluses to prevent hypotension during spinal anesthesia for cesarean delivery: a sequential allocation dose-finding study. Anesth Analg.

[7] Vallejo MC, Attaallah AF, Elzamzamy OM (2017). An open-label randomized controlled clinical trial for comparison of continuous phenylephrine versus norepinephrine infusion in prevention of spinal hypotension during cesarean delivery. Int J Obstet Anesth.

[8] Chen X, Qian X, Fu F (2010). Intrathecal sufentanil decreases the median effective dose (ED50) of intrathecal hyperbaric ropivacaine for cesarean delivery. Acta Anaesthesiol Scand.

[9] Zheng J, An X, Qian JY (2020). Research letter: ED(90) of phenylephrine prophylactic bolus dose to prevent maternal hypotension during cesarean delivery. J Clin Anesth.

[10] Loubert C, Gagnon PO, Fernando R (2017). Minimum effective fluid volume of colloid to prevent hypotension during cesarean section under spinal anesthesia using a prophylactic phenylephrine infusion: An up-down sequential allocation study. J Clin Anesth.

[11] Weber F, Wulf H, el Saeidi G (2003). Premedication with nasal s-ketamine and midazolam provides good conditions for induction of anesthesia in preschool children. Can J Anaesth.

[12] Timm C, Linstedt U, Weiss T (2008). Sympathomimetic effects of low-dose S(+)-ketamine. Effect of propofol dosage. Anaesthesist.

[13] Pees C, Haas NA, Ewert P (2003). Comparison of analgesic/sedative effect of racemic ketamine and S(+)-ketamine during cardiac catheterization in newborns and children. Pediatr Cardiol.

[14] Smith-Nielsen J, Matthey S, Lange T (2018). Validation of the Edinburgh Postnatal Depression Scale against both DSM-5 and ICD-10 diagnostic criteria for depression. BMC Psychiatry.

[15] Bhat R, Santhosh MC, Annigeri VM (2016). Comparison of intranasal dexmedetomidine and dexmedetomidine-ketamine for premedication in pediatrics patients: A randomized double-blind study. Anesth Essay Res.

[16] Tang Y, Yang M, Fu F (2020). Comparison of the ED50 of intrathecal hyperbaric ropivacaine coadministered with or without intrathecal dexmedetomidine for cesarean section: a prospective, double-blinded, randomized dose-response trial using up-down sequential allocation method. J Clin Anesth.

[17] Mei Z, Ngan Kee WD, Sheng ZM (2020). Comparative dose-response study of hyperbaric ropivacaine for spinal anesthesia for cesarean delivery in singleton versus twin pregnancies. J Clin Anesth.

[18] Pace NL, Stylianou MP (2007). Advances in and limitations of up-and-down methodology: a précis of clinical use, study design, and dose estimation in anesthesia research. Anesthesiology.

[19] Stylianou M, Proschan M, Flournoy N (2003). Estimating the probability of toxicity at the target dose following an up and-down design. Stat Med.

[20] Xu Z, Xu T, Zhao P, Ma R, Zhang M, Zheng J (2017). Differential roles of the right and left toe perfusion index in predicting the incidence of postspinal hypotension during cesarean delivery. Anesth Analg.

[21] Toyama S, Kakumoto M, Morioka M (2013). Perfusion index derived from a pulse oximeter can predict the incidence of hypotension during spinal anesthesia for cesarean delivery. Br J Anaesth.

[22] Trimmel H, Helbok R, Staudinger T (2018). S(+)-ketamine current trends in emergency and intensive care medicine. Wien Klin Wochenschr.

[23] Bornemann-Cimenti H, Wejbora M, Michaeli K (2016). The effects of minimal-dose versus low-dose s-ketamine on opioid consumption, hyperalgesia, and postoperative delirium: a triple-blinded, randomized, active- and placebo-controlled clinical trial. Minerva Anestesiol.

[24] Suppa E, Valente A, Catarci S (2012). A study of low-dose S-ketamine infusion as "preventive" pain treatment for cesarean section with spinal anesthesia: benefits and side effects. Minerva Anesthesiol.

[25] Swainson J, Thomas RK, Archer S (2019). Esketamine for treatment resistant depression. Expert Rev Neurother.

[26] Corriger A, Pickering G (2019). Ketamine and depression: a narrative review. Drug Des Devel Ther.

[27] Yang C, Shirayama Y, Zhang JC (2015). R-ketamine: a rapid-onset and sustained antidepressant without psychotomimetic side effects. Transl Psychiatry.

[28] Xu Y, Li Y, Huang X (2017). Single bolus low-dose of ketamine does not prevent postpartum depression: a randomized, double-blind, placebo-controlled, prospective clinical trial. Arch Gynecol Obstet.

[29] Cristea LA, Naudet F (2019). US Food and Drug Administration approval of esketamine and brexanolone. Lancet Psychiatry.

[30] Sen S, Ozmert G, Aydin ON (2005). The persisting analgesic effect of low-dose intravenous ketamine after spinal anesthesia for cesarean section. Eur J Anaesthesiol.

[31] Menkiti ID, Desalu I, Kushimo OT (2012). Low-dose intravenous ketamine improves postoperative analgesia after cesarean delivery with spinal bupivacaine in African parturients. Int J Obstet Anesth.

